# Succession and Fermentation Products of Grass Carp (*Ctenopharyngodon idellus*) Hindgut Microbiota in Response to an Extreme Dietary Shift

**DOI:** 10.3389/fmicb.2017.01585

**Published:** 2017-08-21

**Authors:** Yao Tong Hao, Shan Gong Wu, Fan Xiong, Ngoc T. Tran, Ivan Jakovlić, Hong Zou, Wen Xiang Li, Gui Tang Wang

**Affiliations:** ^1^Key Laboratory of Aquaculture Disease Control, Ministry of Agriculture, and State Key Laboratory of Freshwater Ecology and Biotechnology, Institute of Hydrobiology, Chinese Academy of Sciences Wuhan, China; ^2^Ocean College of Hebei Agricultural University Qinhuangdao, China; ^3^University of Chinese Academy of Sciences Beijing, China; ^4^Bio-Transduction Lab, Wuhan Institute of Biotechnology Wuhan, China

**Keywords:** gut microbiota, SCFAs, high-protein diet, high-fiber diet, freshwater fish

## Abstract

Dietary intake affects the structure and function of microbes in host intestine. However, the succession of gut microbiota in response to changes in macronutrient levels during a long period of time remains insufficiently studied. Here, we determined the succession and metabolic products of intestinal microbiota in grass carp (*Ctenopharyngodon idellus*) undergoing an abrupt and extreme diet change, from fish meal to Sudan grass (*Sorghum sudanense*). Grass carp hindgut microbiota responded rapidly to the diet shift, reaching a new equilibrium approximately within 11 days. In comparison to animal-diet samples, *Bacteroides*, Lachnospiraceae and Erysipelotrichaceae increased significantly while *Cetobacterium* decreased significantly in plant-diet samples. *Cetobacterium* was negatively correlated with *Bacteroides*, Lachnospiraceae and Erysipelotrichaceae, while *Bacteroides* was positively correlated with Lachnospiraceae. Predicted glycoside hydrolase and polysaccharide lyase genes in *Bacteroides* and Lachnospiraceae from the Carbohydrate-Active enZymes (CAZy) database might be involved in degradation of the plant cell wall polysaccharides. However, none of these enzymes was detected in the grass carp genome searched against dbCAN database. Additionally, a significant decrease of short chain fatty acids levels in plant-based samples was observed. Generally, our results suggest a rapid adaption of grass carp intestinal microbiota to dietary shift, and that microbiota are likely to play an indispensable role in nutrient turnover and fermentation.

## Introduction

The vertebrate gastrointestinal tract is populated by a complex bacterial community, which has now been considered to be an integral component of the host, and exerts a strong influence on host biology (Ley et al., 2008). Among its many known functions, the gut microbiome plays an important role in facilitating the digestion and assimilation of indigestible dietary components, mainly plant-derived polysaccharides (Flint et al., 2008). Without this microbial fermentation, calories in a diverse array of complex dietary glycans would be unavailable to the host (Martens et al., 2009). Hence, gut bacterial fermentation may improve feed conversion efficiency (Flint et al., 2008). The adaption of intestinal microbiota to different foods has already been relatively well-understood in terrestrial mammals (David et al., 2014). Long-term dietary intake also influences the structure and function of numerous microorganisms residing in the fish gut (Ringø et al., 2006). However, the succession of gut microbiome of fish in response to radical changes in macronutrients, remains unclear.

Grass carp (*Ctenopharyngodon idellus*) is a native Chinese freshwater fish and has now been introduced to more than 100 countries. Grass carp is the largest aquaculture product globally^1^. Larval grass carp feeds on zooplankton (Ni and Wang, 1999). Thereafter, it feeds on aquatic plants by using comblike pharyngeal teeth to grind plant material when the fish is about 4.0 g in weight or 5.0 cm in length (Li, 1981; Cui et al., 1991). Although it is predominantly herbivorous and prefers aquatic plant, the fish may feed on tiny aquatic animals, such as shrimp and small fish, in the absence of aquatic plants (Li, 1981). Elucidating the roles of gut microbiota in feed digestion is essential for understanding the adaption of host to extreme diet change. Previous studies of the intestinal microbiota in grass carp have focused mainly on characterizing bacterial composition, abundance and diversity under a certain feeding regime (Wu et al., 2012). Some studies based on bacterial isolation and enzyme assays have confirmed the presence of bacterial activities of cellulase, amylase, protease and lipase in the intestine (Feng et al., 2008; Li et al., 2016). Recently, comparative transcriptomics has revealed that the bacterial transcriptomes in gut are influenced by dietary nutrients and that the grass carp intestinal microbiome functions in carbohydrate turnover and fermentation, which likely provides energy to both the host and microbiota (Wu et al., 2015). However, so far, the precise succession of grass carp gut microbiota in response to changing dietary conditions has not been determined. A continuous monitoring of fish gut microbiota may disclose a dynamic responsive process of bacterial communities to shifts in diet.

Fecal short-chain fatty acids (SCFAs) are the major end products of bacterial fermentation of polysaccharides, oligosaccharides, proteins, peptides and glycoprotein precursors in the intestine (Cummings and Macfarlane, 1991). Several lines of evidence have indicated that some herbivorous marine fishes have high levels of SCFAs in their hindgut and appear to rely on hindgut fermentation for meeting their nutritional requirements (Mountfort et al., 2002). However, herbivorous freshwater fishes tend to have shorter gut transit times and thus also lower SCFA levels in the gut (German, 2009). Determining SCFAs in grass carp intestine will provide an insight into the importance of gut bacterial community in energy harvest and offer a direct evidence of microbial fermentation.

In this study, we have determined the succession and metabolic products of grass carp gut microbiota in response to extreme diet-type change: a transition from animal-based diet (fish meal) to plant-based diet (*Sorghum sudanense*). Our goals were to elucidate the adaption of intestinal microbiota to different diets and accompanying changes in fermentation products.

## Materials and Methods

### Experimental Fish and Feed

Juvenile fish were purchased commercially and raised in an artificial pond (depth ≈ 2.0 m, surface area ≈ 2000 m^2^) in Wuhan, China. From July 2012, they were fed animal-based diet (granular fish meal) at 4% of their body weight per day for almost 75 days, until September 16th. Then, from September 17th to October 19th 2012, they were fed a plant-based diet (*Sorghum sudanense*) to satiation once a day (09:30 a.m.). Feed chemical composition of fresh Sudan grass and granular fish meal (in %) is shown in **Table 1** (Wu et al., 2015). Average daily water temperatures were 26.6°C in September and 22.0°C in October.

**Table 1:** Chemical composition of the experimental diets (% dry matter).

Proximate chemical composition	Granular fish meal	Sudan grass
Crude fiber	0.49	29.00
Crude Protein	62.76	10.37
Crude lipid	5.45	3.75
Crude ash	11.09	6.33

### Sample Collection and DNA Extraction

The last day of fish meal feeding (September 16th) was recorded as day 0. Sampling was undertaken between September 16th and October 19th, on days 0, 1, 3, 7, 11, 19, 25, and 33 (each at 3:00 pm). Each time, three specimens were caught randomly by net, euthanized in buffered MS-222 at 100 mg/L concentration, measured (weight and length) and immediately dissected. The hindgut (from the last crook of gut to the anus) was divided from the whole intestine and hindgut content was taken out as a sample. Weight and length of the sampled specimens ranged from 0.8 to 1.2 kg and 32.5 to 45.0 cm, respectively. All samples were frozen immediately in a freezer (-20°C) and brought to the laboratory within 24 h for storage at -80°C. Approximately 200 mg of hindgut content were used for the extraction of genomic DNA (QIAamp^®^ DNA Stool Mini Kit, Qiagen, Germany). Sterile zirconia beads were added to the samples to improve extraction yield and quality of the community DNA (Yu and Morrison, 2004). Two separate extractions of each sample were performed and pooled together to avoid bias. The extracted DNA samples were stored at -20°C. To determine the stability of gut bacterial community before the change in diet, samples of grass carp fed on fish meal from Wu et al. (2015) were included in the present study; the fish were fed with fish meal for more than 2 months and reared at the same fish farm as our samples.

### 16S rDNA Gene-Based Pyrosequencing

The V1–V3 region of the 16S rDNA gene was amplified with barcoded primers (8F/533R) (Wu et al., 2012). Replicated PCR products of each sample were pooled and separated by 2% agarose gel electrophoresis, then purified with a DNA gel extraction kit (Axygen, China). Prior to sequencing, the concentrations of purified amplicon DNA were determined using a QuantiFluorTM-ST Fluorometer (Promega, Madison, WI, United States). Equal amounts of the PCR products were mixed together and sequenced on a 454 GS FLX + platform (Roche Applied Science, Indianapolis, IN, United States) at Majorbio Bio-Pharm Technology Co., Ltd., Shanghai, China.

### Sequence Data Processing

Raw sequences were processed using Seqcln^2^ and Mothur^3^ software packages (Chen et al., 2007; Schloss et al., 2009). Sequences were removed from the analyses if they did not contain the primer sequence, an uncorrectable barcode, any ambiguous characters, a mean quality score < 25, or length < 400 nt. The remaining sequences were assigned to each sample by examining the 10 nt unique barcode. The valid sequences were reduced to a subset of unique sequences in order to eliminate redundancy and reduce computation time. This reduced dataset was aligned and compared to the Bacterial SILVA database (version SSU111) by kmer searching^4^. The chimeric sequences were removed by UCHIME (Edgar et al., 2011). The sequences were clustered into operational taxonomic units (OTUs) using the furthest neighbor method^5^ with the identity threshold set at 97%, while OTUs with only one sequence were removed. The taxonomic assignment of each OTU sequence was performed using Mothur^6^ and the Ribosomal Database Project (RDP) classifier (Wang et al., 2007). All the DNA-Seq datasets were submitted to the GenBank Short Read Archive under the accession number SRX1056223.

### qPCR Assays

The quantitative PCR assays targeted prokaryotes, bacteria, archaea, and bacterial subgroups. Details of the standards, PCR conditions, and references are provided in Supplementary Text S1 and Table S1. The number of copies of the 16S rDNA gene per gram of gut content was calculated as follows: N = Q/2 × D × (50 μL)/0.2 g, Q is the detected copy number from 2 μL of diluted template, D is the dilution factor, 50 μL is the elution volume in genomic DNA extraction, and 0.2 g is the wet weight of gut content used for DNA extraction.

### Prediction of Possible Glycoside Hydrolases (GHs) and Polysaccharide Lyases (PLs)

The GH and PL families in the genomes of *Bacteroides* and grass carp were analyzed. The GH and PL genes of *Bacteroides* spp. were collected directly from the Carbohydrate-Active enZymes (CAZy) database (Cantarel et al., 2009). The *Bacteroides* spp. used in this study are listed in Supplementary Text S2. The genome of grass carp was searched against dbCAN database^7^ (Yin et al., 2012; Wang et al., 2015) with default parameters. GH families of Lachnospiraceae in this study were from Biddle et al. (2013). The peptide enzymes of *Cetobacterium somerae* ATCC BAA-474 were analyzed based on the UniProt Knowledgebase (UniProtKB^8^).

### Predictive Functional Profiling of Microbial Communities

PICRUSt (phylogenetic investigation of communities by reconstruction of unobserved states) 1.0.0 was used to predict the functional composition of each sample (Langille et al., 2013). PICRUSt is a computational approach to predict gene family abundance (e.g., the metagenome) using marker gene (usually 16S rRNA) data. In brief, 16S rRNA genes were clustered into OTUs at the 97% similarity threshold by QIIME (Caporaso et al., 2010). Taxonomy was assigned using the RDP classifier. Then, the OTU abundance was normalized automatically using 16S rRNA gene copy numbers from the reference bacterial and archaeal genomes in Integrated Microbial Genomes (IMG) database (Markowitz et al., 2012). Finally, a ‘virtual’ metagenome of clusters of orthologous groups (COGs) with their abundances were produced based on the previous normalized OTU table using the default settings in PICRUSt (Tatusov et al., 1997; Langille et al., 2013).

### Short-Chain Fatty Acid (SCFA) Analysis

Short-chain fatty acids of grass carp hindgut samples were extracted and determined using gas chromatography as previously described (Schneider et al., 2006; Zhao et al., 2006). In brief, 200 mg of the sample was suspended in 1.6 mL sterile distilled water with an internal standard hexanoic acid (5 μL). 50% aqueous sulfuric acid (0.4 mL) and diethyl ether (2 mL) were then added. The sample was mixed for 45 min with an orbital shaker and centrifuged for 5 min at 3000 rpm at room temperature. Concentrations of the SCFAs in the supernatant were determined using an Agilent 7890A gas chromatograph equipped with flame ionization detection (Agilent, United States). A fused-silica capillary column (30 m, 0.52 mm, 0.50 mm) with a free fatty acid phase (DB-FFAP 125-3237, J&W Scientific, Agilent Technologies Inc.) was used.

### Statistical Analyses

Alpha-diversity (including ACE, Chao1, Shannon, and Simpson estimators) was calculated using Mothur. Distances between microbial communities in different samples were calculated using the weighted UniFrac beta-diversity metric via Mothur. Principal coordinates analysis (PCoA) was used to visualize the pairwise UniFrac distances among samples through R.^9^ Heat maps showing relative taxonomic abundance were generated in R. The shared GH and PL families between grass carp and bacterial groups of *Bacteroides* spp. and Lachnospiraceae were presented using a Venn diagram. The relationships among the present samples and those fed on fish meal from Wu et al. (2015) were determined using cluster analysis based on Bray-Curtis distance. Permutational multivariate analyses of variance (PERMANOVA) were performed by PAST 2.16 (Hammer et al., 2013) to assess the significance of differences in bacterial community structure among groups of hindgut samples. Statistical significance was set at a *P*-value of <0.05. Statistically significant differences were compared using a one-way ANOVA followed by Tukey’s *post hoc* multiple comparison tests.

To understand the correlations among different OTUs within a community or different communities, molecular ecological networks based on Random Matrix Theory (RMT) methods (Deng et al., 2012) were constructed following the MENA pipeline^10^ with default parameters. OTUs from 24 samples were analyzed. Any OTU presented in <12 samples was excluded. Modules were detected using fast greedy modularity optimization. Cytoscape software 3.2.1 was used to visualize the network graphs (Shannon et al., 2003). The significance of correlation between any two OTUs or bacterial groups was evaluated using the Pearson correlation coefficient (r).

## Results

**FIGURE 1:**
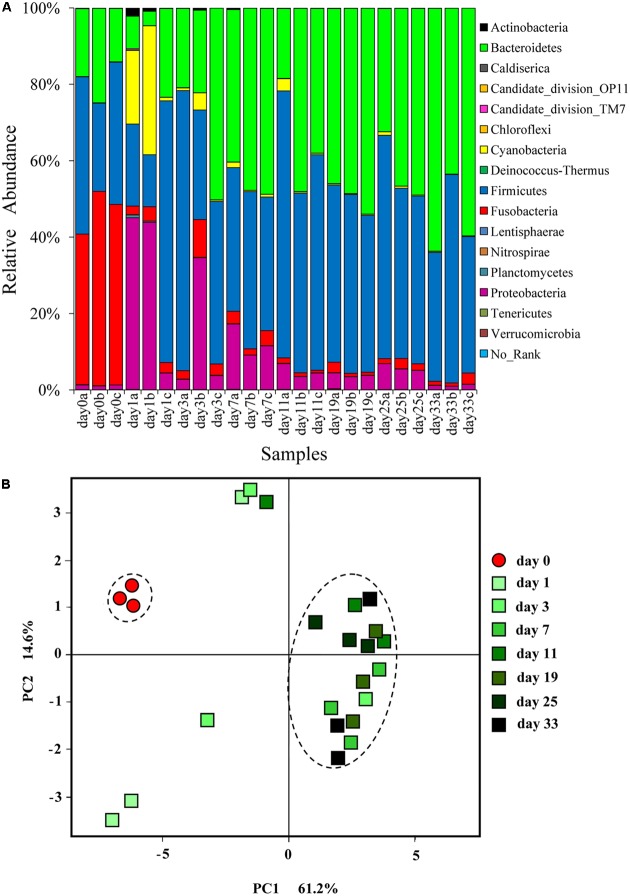
**(A)** Relative abundance of different bacterial phyla in the hindgut of grass carp fed on fish meal (day 0) and Sudan grass (day 1–33). Sequences that cannot be classified into any known group are listed as “No-Rank.” **(B)** Changes in grass carp hindgut microbiota in response to dietary shift. Principal coordinates analysis (PCoA) plot showing the microbial community differences among different time-point samples. Pairwise community distances are determined using the weighted UniFrac algorithm.

A total of 447,009 high-quality sequences with an average length of 494.5 nt, belonging to 5494 OTUs were obtained from 24 hindgut content samples (Supplementary Table S2). The number of sequences ranged from 14,782 to 22,420 per sample and Good’s coverages ranged from 96.0 to 98.8%. Here, 16 bacterial phyla were detected (**Figure 1A**). Most of the sequences detected in grass carp hindgut were classified as Firmicutes (43.4%) and Bacteroidetes (35.3%). Proteobacteria (10.2%), Fusobacteria (7.3%), and Cyanobacteria (3.3%) were also abundant. Bacterial 16S rRNA genes in Sudan grass were successfully amplified and almost 90% of them were classified as Cyanobacteria (Supplementary Figure S1). As the origin of these Cyanobacteria is likely to be from the chloroplast DNA, and as no bacterial DNA was found in the animal-based diet (fish meal), we can conclude that both animal and plant diets did not contain significant amounts of bacteria.

### Changes in the Diversity and Structure of Intestinal Microbiota in Response to Different Dietary Nutrients

Cluster analysis at the phylum level showed that day 0 samples and animal-diet samples from Wu et al. (2015) formed a single cluster, clearly distinct from plant-diet samples (Supplementary Figure S2). PERMANOVA analysis did not find a significant difference (*F* = 13.6, *P* = 0.101) in bacterial communities between day 0 samples and animal-diet samples from Wu et al. (2015). These results indicate that microbial communities were stable before the dietary shift.

**FIGURE 2:**
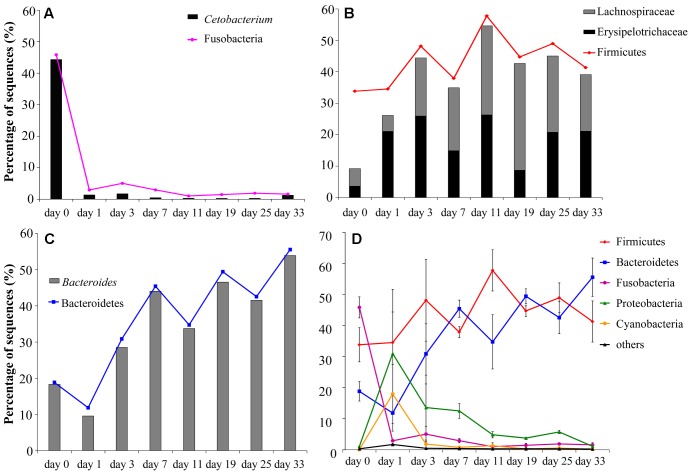
Changes in relative abundances of the main bacterial communities (shown as means of the values of three individual fishes) in the grass carp hindgut after a sudden transition from animal-based diet (fish meal, day 0) to plant-based diet (Sudan grass, day 1–33): **(A)** Fusobacteria and *Cetobacterium*; **(B)** Lachnospiraceae, Erysipelotrichaceae and Firmicutes; **(C)** Bacteroidetes and *Bacteroides*; **(D)** comparison of the changes in the five main phyla, with standard deviation values shown as error bars.

In comparison to day 0 samples, day 3–33 samples exhibited significantly higher Ace and Chao indices (*P* < 0.05), but only the day 7 samples exhibited a significantly higher diversity according to Shannon and Simpson indices (**Table 2**). PCoA based on UniFrac distances showed that day 0 samples formed a cluster, clearly separated from plant-diet samples, while only the plant-diet samples 11 days later clustered together (**Figure 1B**). A substantial change in membership and structure in the hindgut bacterial community occurred on the first day of the dietary shift, followed by a period of instability during the first 11 days (**Figures 1B**, **2**). PERMANOVA indicated that microbial communities before and after the diet shift were significantly different (*F* = 10.1, *P* < 0.05). Within the plant-diet samples, microbial communities of day 1–7 samples differed significantly from those of day 11–33 samples (*F* = 4.1, *P* < 0.05), but the microbial structures among day 11–33 samples were not significantly different (*P* > 0.05).

**Table 2:** Summary of diversity indices and coverage estimators of different time-point samples.

Sample ID (*n* = 3)	Coverage (%)	α-diversity				β-diversity
		Shannon	Simpson	Ace	Chao	UniFrac distance^r^
day 0	97.70 ± 0.06	3.14 ± 0.21	0.16 ± 0.02	1751 ± 226	1277 ± 126	0.09 ± 0.01
day 1	98.25 ± 0.78	3.61 ± 0.60	0.12 ± 0.08	1741 ± 1041	1419 ± 583	0.48 ± 0.01
day 3	97.23 ± 0.77	3.56 ± 0.65	0.13 ± 0.12	2917 ± 752^∗^	2006 ± 370^∗^	0.48 ± 0.02
day 7	97.28 ± 0.20	3.84 ± 0.21^∗^	0.06 ± 0.01^∗^	2546 ± 263^∗^	1877 ± 143^∗^	0.54 ± 0.01
day 11	96.91 ± 0.45	3.47 ± 0.13	0.11 ± 0.03	2833 ± 445^∗^	1990 ± 266^∗^	0.52 ± 0.01
day 19	96.99 ± 0.17	3.33 ± 0.15	0.11 ± 0.02	2810 ± 312^∗^	1876 ± 121^∗^	0.53 ± 0.01
day 25	96.76 ± 0.70	3.42 ± 0.13	0.10 ± 0.01	3077 ± 492^∗^	2060 ± 293^∗^	0.54 ± 0.01
day 33	96.31 ± 0.23	3.35 ± 0.09	0.12 ± 0.00	3509 ± 310^∗^	2292 ± 203^∗^	0.60 ± 0.01

^∗^*Significant difference from day 0 samples (*P* < 0.05). ^*r*^UniFrac distance relative to day 0; for day 0, among day 0 samples only*.

### Temporal Analysis of the Reconfiguration of Bacterial Taxa after the Dietary Shift

At the beginning of the experiment (day 0), the hindgut of grass carp was dominated by Fusobacteria (45.8 ± 5.9%), Firmicutes (33.8 ± 9.5%) and Bacteroidetes (18.8 ± 5.5%). On day 1 of the dietary shift, Firmicutes (34.5 ± 29.7%) and Proteobacteria (31.0 ± 23.1%) were the most abundant groups, with Cyanobacteria (18.0 ± 16.5%) and Bacteroidetes (11.9 ± 10.1%) also relatively abundant. Relative abundance of Fusobacteria decreased significantly in the day 1 sample (*P* < 0.05, 2.9 ± 0.8%) and remained low until the end of the experiment. *Cetobacterium somerae* comprised about 96.7% Fusobacteria (**Figure 2A**). In comparison to day 0 samples, Bacteroidetes were significantly more abundant in day 3–33 samples (*P* < 0.05). From day 11, Firmicutes and Bacteroidetes comprised more than 90% of the microbial community (**Figures 1**, **2D** and Supplementary Figure S3). Standard deviation values of different bacterial phyla among the three samples taken at each time point were as follow: low on day 0, very high on day 1, and then rapidly decreasing afterward (**Figure 2D**).

The relative abundance of Firmicutes was not significantly different between animal- and plant-diet samples (*F* = 1.4, *P* = 0.242), but the proportions of Lachnospiraceae and Erysipelotrichaceae both Firmicutes increased significantly (*F* = 4.5, *P* < 0.05; *F* = 4.4, *P* < 0.05, respectively) in plant-diet samples: 9.21% on day 0, 26.1–54.6% on day 1–33 (**Figure 2B**). All sequences from Bacteroidaceae, the main bacterial group in Bacteroidetes belonged to *Bacteroides*. The relative abundance of *Bacteroides* rapidly decreased between day 0 and 1 samples (18.3 ± 5.5% to 9.6 ± 11.5%), but then again significantly increased afterward (28.5% to 53.9%; all *P* < 0.05) (**Figure 2C**). As opposed to the high percentages observed in plant-diet intestinal samples, the relative abundance of *Bacteroides*, Lachnospiraceae and Erysipelotrichaceae found in Sudan grass was less than 0.6%.

Further analyses indicated that 14 OTUs were shared by all samples, six of which belonged to *Bacteroides*, five to Lachnospiraceae, and the remaining three OTUs belonged to Erysipelotrichaceae, Clostridiales, and *Aeromonas* (Supplementary Figure S4). The relative abundance of these shared OTUs in all sequences varied from 27.6 to 73.9% at different time points (57.4% of total sequences in all samples). With the exception of the day 1 sample, the proportion of these 14 OTUs was significantly higher in plant-diet samples than on day 0 (*P* < 0.05).

### Quantification of the Intestinal Microbiota by qPCR

The counts of 16S rRNA gene copies of prokaryotes and bacteria in the day 0 (animal-diet) sample (2.9 × 10^10^ and 7.6 × 10^9^ copies per gram of gut content, respectively) were both significantly higher than the counts in day 1–7 samples and day 11–33 samples (*P* < 0.05). However, no significant differences were observed between day 1–7 and day 11–33 samples (*F* = 1.8, *P* = 0.099 for prokaryotes; *F* = 2.1, *P* = 0.055 for bacteria). 16S rRNA gene copy counts in day 1– 33 samples ranged from 5.3 × 10^8^ to 5.3 × 10^9^ copies (prokaryotes) and 2.6 × 10^8^ to 1.1 × 10^9^ copies (bacteria) per gram of gut content. The highest number of archaea 16S rRNA gene copies (6.2 × 10^3^ to 2.3 × 10^4^ copies) was also observed on day 0, but the following decrease was not significant (Supplementary Figure S5). The ratios of *Bacteroides*/Bacteroidetes and Lachnospiraceae/Firmicutes measured by qPCR were consistent with the results of 454 pyrosequencing (Supplementary Figure S6).

### Predicted GH and PL Families in Host and Intestinal Microbiota

Venn diagram showed that 18 GH families and one PL (PL15) family were shared among grass carp and bacterial groups Lachnospiraceae and *Bacteroides* (Supplementary Figure S7). Among these, GH5 encoded cellulase in bacteria, but β-glucuronidase in grass carp; while PL15 encoded pectinase in bacteria, but dermatan-sulfate epimerase in grass carp. GH9 and GH10 were typical cellulase and xylanase, respectively, and were only found in intestinal bacterial genomes (Supplementary Figure S7A). The GH28, GH88, GH105, PL1, PL9, PL10, and PL11 gene families only detected in intestinal microbiota also encoded pectinases (Supplementary Figure S7). The most abundant GH gene families in *Bacteroides* were GH2 and GH43, while the most abundant PL families were PL1 and PL11 (Supplementary Figure S8). The genes encoding endopeptidase, carboxypeptidase, aminopeptidase, and dipeptidyl-peptidase were found in *C. somerae* ATCC BAA-474 (Table S3).

### Functions of Microbial Communities Inferred by Predictive COGs

**FIGURE 3:**
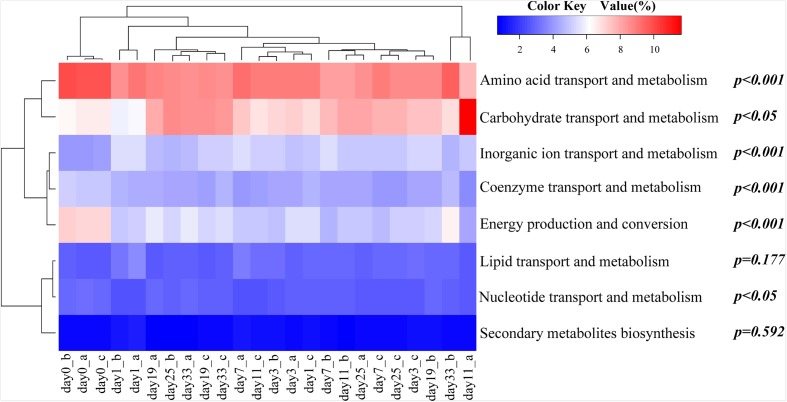
Heat map showing relative abundances of clusters of orthologous group (COG) categories predicted by PICRUSt. The relationship among specimens is determined by the complete clustering method with Bray-Curtis distance. In the heat map, the red and blue colors indicate high and low relative abundance, respectively. The *P*-values exhibit statistical differences in relative abundances of COG categories between animal-diet samples and day 11–33 plant-diet samples, where *P* < 0.05 was chosen as statistically difference. a, b, and c represent different samples on the same day.

A ‘virtual’ metagenome of COGs with their abundances, was predicted based on the presence of 16S rDNAs by PICRUSt. PICRUSt-prediction revealed significantly more abundant genes associated with amino acid and energy metabolism in animal-diet samples (day 0) in comparison to day 11–33 plant-diet samples (*F* = 23.0, *P* < 0.001; *F* = 34.4, *P* < 0.001) (**Figure 3**). On the contrary, carbohydrate transport and metabolism genes were more abundant in day 11–33 plant-diet samples (*F* = 5.7, *P* < 0.05) (**Figure 3**). Genes associated with lipid metabolism did not change significantly with the dietary shift (*F* = 2.0, *P* = 0.177) (**Figure 3**).

### Correlations among Microbial Groups

**FIGURE 4:**
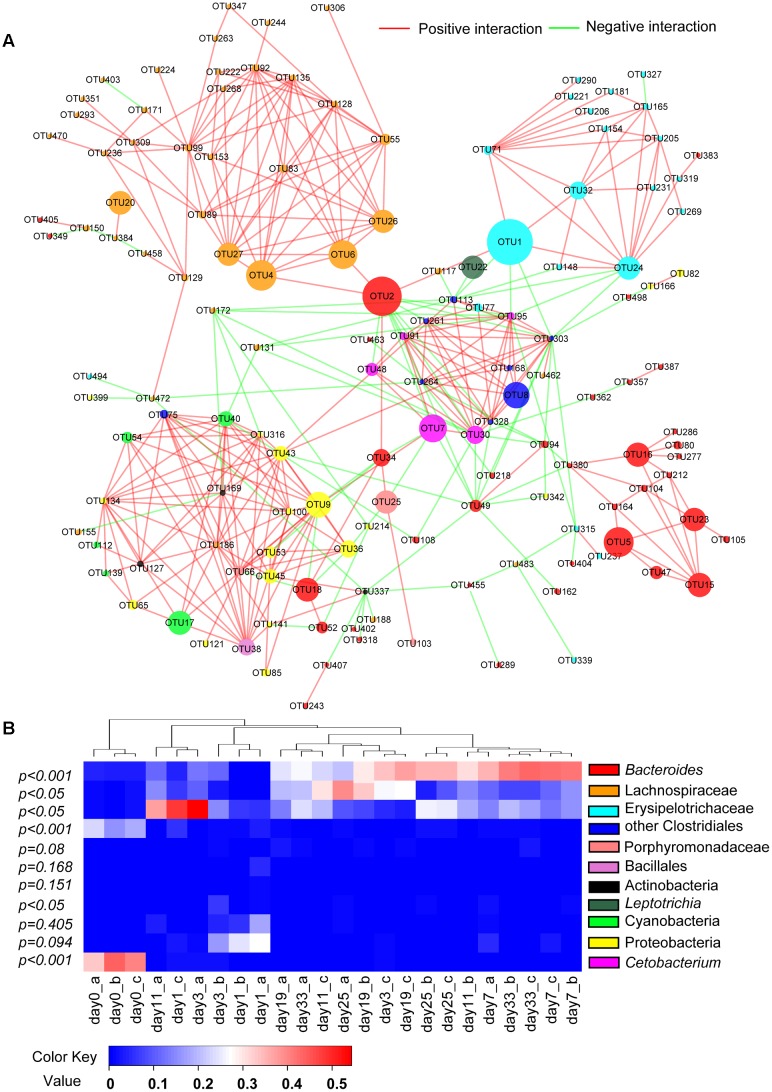
Correlations and changes in relative abundance of microbial communities after a sudden transition from animal-based diet (fish meal, day 0) to plant-based diet (Sudan grass, day 1–33) in grass carp. **(A)** Molecular ecological network (MEN) of operational taxonomic units (OTUs). The module structure of network graph is based on the fast greedy modularity optimization method. Each node signifies an OTU, which can correspond to a microbial population. The colors of the nodes indicate different bacterial groups. The sizes of the nodes indicate different abundances in total sequences. A green edge indicates a negative correlation between two individual nodes, and a red edge indicates a positive correlation. **(B)** Heat map of specimens showing relative abundance of each bacterial group presented in MENA. The relationship among specimens is determined by the complete clustering method with Bray-Curtis distance. In the heat map, red color means higher relative abundance whereas blue color signifies lower relative abundance. The *P*-values exhibit statistical differences in bacterial abundances between animal-diet samples and day 11–33 plant-diet samples. *P* < 0.05 indicates significant difference.

After excluding all OTUs present in <12 samples, total of 141 OTUs or 64.8% of all sequences were selected through MENA analysis. Among them, bacterial groups of *Bacteroides*, Lachnospiraceae and Erysipelothrichaceae were significantly less abundant in animal-diet samples than in days 11–33 plant-diet samples (*F* = 22.8, *P* < 0.001; *F* = 6.8, *P* < 0.05; *F* = 9.6, *P* < 0.05, respectively), while *Cetobacterium* and some Clostridiales bacteria were significantly more abundant in animal-diet samples (*F* = 700.1, *P* < 0.001; *F* = 341.2, *P* < 0.001, respectively) (**Figure 4**). Almost all correlations among OTUs from the same taxonomic group, such as *Bacteroides*, *Cetobacterium*, Lachnospiraceae and Erysipelothrichaceae, were positive (**Figure 4**) (*P* < 0.05). However, correlations among different bacterial taxonomic groups varied: *Cetobacterium* was negatively correlated with *Bacteroides* (*r* = -0.512, *P* = 0.005), Lachnospiraceae (*r* = -0.403, *P* = 0.025) and Erysipelothrichaceae (*r* = -0.512, *P* = 0.005). OTU2 (*Bacteroides*), which was particularly abundant, exhibited a positive correlation with Lachnospiraceae (*r* = 0.464, *P* = 0.001) (**Figure 4**).

### SCFA Levels in the Hindgut

Total SCFAs in hindgut contents varied from 2.41 to 7.68 mM (**Table 3**). Differences in SCFA concentrations between animal-diet samples and plant-diet samples were significant (*F* = 43.1, *P* < 0.001). Acetic acid was the dominant SCFA, followed by butyric and propionic acids. Propionic acid was less abundant than butyric acid in both animal-diet (*F* = 0.3, *P* = 0.62, non-significant) and plant-diet (*F* = 47.8, *P* < 0.001, highly significant) samples. The total SCFA concentrations of acetate, propionate, and butyrate decreased almost 50% after the change from animal-based diet to plant-based diet. The ratio of acetate to propionate increased significantly after the dietary shift (*F* = 3.2, *P* < 0.05). In addition, valeric acid, iso-butyric acid and iso-valeric acid levels were significantly lower than those of acetic, propionic, and butyric acids regardless of the type of feed (*P* < 0.05). The analysis of standards is shown in Supplementary Figure S9.

**Table 3:** Major SCFA concentrations in hindgut content samples (mmol/kg wet weight).

Sample ID (*n* = 3)	Acetic	Propionic	Iso-butyric	Butyric	Total
Day 0	4.50 ± 0.45	1.58 ± 0.51	0.23 ± 0.01	2.07 ± 0.11	7.68 ± 1.06
Day 1	1.60 ± 1.07^∗^	0.22 ± 0.23^∗^	0.11 ± 0.11	0.48 ± 0.61	2.41 ± 2.02^∗^
Day 3	2.43 ± 0.44^∗^	0.10 ± 0.03^∗^	0.02 ± 0.03^∗^	0.82 ± 0.30	3.37 ± 0.75^∗^
Day 7	2.35 ± 0.31^∗^	0.18 ± 0.06^∗^	0.02 ± 0.01^∗^	0.79 ± 0.33	3.349 ± 0.59^∗^
Day 11	2.92 ± 0.38^∗^	0.12 ± 0.12^∗^	0.03 ± 0.04^∗^	0.75 ± 0.20	3.82 ± 0.38^∗^
Day 19	2.58 ± 0.40^∗^	0.17 ± 0.10^∗^	0.17 ± 0.01	0.82 ± 0.07	3.75 ± 0.57^∗^
Day 25	3.14 ± 0.41	0.20 ± 0.10^∗^	0.12 ± 0.10	0.75 ± 0.24	4.21 ± 0.66^∗^
Day 33	2.10 ± 0.28^∗^	0.45 ± 0.10^∗^	0.03 ± 0.01^∗^	0.33 ± 0.11^∗^	2.91 ± 0.48^∗^

^∗^*Significant difference from day 0 samples (*P* < 0.05)*.

## Discussion

### Succession of the Intestinal Microbial Composition and Function in Response to Different Diets

Gut microbial communities changed rapidly when the diet shifted, underwent a short period of high instability, and then began to stabilize again since day 11, approximately. Exceptionally high standard deviation values among day 1 samples may have been caused by the individual differences in food consumption on the day of the diet change. The rapid change followed by the subsequent stabilization of microbial structure in grass carp hindgut is consistent with the observations in terrestrial mammals undergoing diet changes (Turnbaugh et al., 2009; David et al., 2014). Grass carp fed fish meal harbored hindgut microbial communities dominated by Fusobacteria and Firmicutes. Fusobacteria is commonly distributed in the intestine of omnivorous and carnivorous freshwater fishes (Sugita et al., 1991; Wu et al., 2010). Grass carp fed on Sudan grass harbored hindgut microbial communities dominated by Firmicutes and Bacteroidetes, which is similar to both mammalian herbivores and some marine herbivorous fish (Bergman, 1990; Sullam et al., 2012). The finding of fourteen OTUs common to all samples (ranging from 27.6 to 73.9%; Supplementary Figure S4) suggests that, similar to rainbow trout (*Oncorhynchus mykiss*) (Wong et al., 2013), core microbiota, comprising of a minority of bacterial species, exists in grass carp digestive system, and likely plays an important role in food digestion.

A significant increase in species richness from animal-diet samples to plant-diet samples (**Table 2**) is congruent with results reported in mammals, where carnivores had the lowest, omnivores medium, and herbivores the highest diversity (Ley et al., 2008; Xu and Knight, 2015). *Cetobacterium somerae*, known to be in a positive correlation with the production of acetic and propionic acids through peptone and glucose fermentation (Tsuchiya et al., 2008), comprised 96.7% of Fusobacteria sequences (**Figure 2**). Numerous gene families associated with protein digestion (peptidases) present in *C. somerae* genome (Supplementary Table S3) could indirectly explain the high abundance of this microbe in animal-diet samples. Higher proportions of *Bacteroides*, Lachnospiraceae and Erysipelotrichaceae found in plant-diet samples have been also associated with low-energy or high-carbohydrate diets in mammals, including humans as well (Hayashi et al., 2002; Hang et al., 2012; Thoetkiattikul et al., 2013). Predicted *Bacteroides* and Lachnospiraceae genomes contain many genes belonging to CAZyme families (including GH and PL families), which can break down a wide variety of indigestible (for the host) polysaccharides and play important roles in the fermentation of dietary fibers (El Kaoutari et al., 2013; Yutin and Galperin, 2013). Many members of the cellulase (GH5 and GH9), hemicellulase (GH10), and pectinase (GH28, GH88, GH105, PL1, PL9, PL10, PL11, and PL15) families present in *Bacteroides* spp. and Lachnospiraceae genomes may be involved in the degradation of the main chain of cell wall polysaccharides: cellulose, xylan and pectin. However, none of the enzymes involved in the degradation of plant cell wall polysaccharides have been detected in grass carp genome searched against dbCAN database yet (Wang et al., 2015), indirectly suggesting that gut microbiota are likely to be indispensable for digestion of plant cell wall polysaccharides in the digestive system of grass carp. Along with *Bacteroides* and Lachnospiraceae, the relative abundance of Erysipelotrichaceae also increased 1.4- to 6.3-fold after the dietary shift, which could be attributed mainly to the increase in OTU1. Search against the GenBank database indicates that OTU1 should be *Clostridium* XVIII (Supplementary Figure S10) (Yutin and Galperin, 2013), which is positively associated with high-carbohydrate diet (Hayashi et al., 2002; Hang et al., 2012).

Previously, a functional analysis of grass carp intestinal metatranscriptome indicated that the G category (carbohydrate transport and metabolism) was positively correlated to the fiber contents in a diet (Wu et al., 2015). Similarly, in this study we have found that bacterial genes associated with carbohydrate transport and metabolism might be more abundant in fish fed Sudan grass diet, whereas genes related to energy and protein metabolism might be more abundant in animal-diet samples, which closely reflects the bacterial compositional differences between the two dietary intestinal samples. In conclusion, the observed increases in the proportions of *Bacteroides*, Lachnospiraceae and Erysipelotrichaceae and decrease in the proportion of *Cetobacterium* are in agreement with the expected intestinal microbiome structure changes associated with the adaption to the switch from a high protein/low fiber diet to a low protein/high fiber diet.

### Molecular Ecological Network Analysis of Microbial Correlations

Network graph indicated that *Cetobacterium* was negatively correlated with *Bacteroides*, Lachnospiraceae and Erysipelotrichaceae, suggesting that these bacterial groups tend to have distinct nutritional requirements. On the other hand, *Bacteroides* showed a positive correlation with Lachnospiraceae, indicating that these two groups undergo synchronous nutritional utilization or metabolic cross-feeding. Primary plant cell wall degraders, for example, may release a wider range of polysaccharides than they utilize, and these become available to bacteria that are less closely adherent to the substrate, or that are planktonic (Dehority, 1991). Studies have shown that non-adherent *Bacteroides* spp. outcompete gram-positive bacteria (such as Firmicutes) for easily hydrolyzable polysaccharide (Macfarlane and Macfarlane, 2006), while the fiber-adherent Lachnospiraceae are uniquely suited to degrade a wide variety of recalcitrant substrates (Brulc et al., 2009). The breakdown of energy-rich complex carbohydrates by gut microbiota creates opportunities for competition and cooperation (Flint et al., 2007).

### Impacts of Diet on Fermentation Products and Bacterial Counts

Short chain fatty acids are bacterial fermentation products. Their concentrations in gut reflect the energy-harvesting capacity of microbiota from diet. The microbial fermentation in the intestine is complex and influenced by many factors, such as intestinal regions, gut transit time, and types of diets (German, 2009; Wu et al., 2015). Grass carp is capable of reducing plant material to 3 mm^3^ particles with its pharyngeal teeth, but it lacks a stomach and pyloric ceca, and has a relatively short intestine, only two times its body length (Ni and Wang, 1999). Resultantly, the transit time of feed in the intestine can be as short as 8 h (Stevens and Hume, 1998), so the products of bacterial metabolism (SCFAs) in the intestine of grass carp are not expected to be highly abundant. The total SCFAs in grass carp hindgut were below 7.68 mM in this study, which is similar to the values reported in some herbivorous minnows (German, 2009), higher than in some wood-eating catfishes (German and Bittong, 2009), and lower than in Nile tilapia (*Oreochromis niloticus*) fed cerel grains (Leenhouwers et al., 2007).

Short chain fatty acid levels decreased significantly after the dietary shift (**Table 3**), and quantitative analyses showed similar trends in total prokaryote and bacteria counts (Supplementary Figure S5). As we also found a positive correlation between the concentration of acetate and the total bacterial count in grass carp gut in our previous study (Hao et al., 2017), it is not unlikely that the lower accessibility (and even indigestibility in some cases) of nutrients in Sudan grass in comparison to fish meal may have resulted in a decline in prokaryote and bacteria counts, leading to a decrease in SCFA levels.

Generally, SCFAs provides 20–30% energy for herbivorous terrestrial animals (Bergman, 1990), but given the relatively low concentrations of SCFAs in grass carp gut, the contribution of these by-products of fermentation to the daily energy requirements of the host is probably limited, which may further explain why grass carp fed on herbal diet need to eat as much as 40–100% of its body weight daily (Ni and Wang, 1999).

## Ethics Statement

No specific permits were required for the described field studies. No specific permissions were required for the artificial pond in Wuhan, Hubei Province, China. It is not privately owned or protected in any way. The field studies did not involve endangered or protected species. This study has been reviewed and approved by the ethics committee of the Institute of Hydrobiology, Chinese Academy of Sciences.

## Author Contributions

SW and GW was the principal investigator and contributed to the study design, acquisition of funding and overseeing the study, interpretation of data and manuscript editing. YH and SW. were in charge of the design, data collection, analysis and interpretation of data and manuscript writing. FX and NT contributed to data analysis and interpretation. IJ was in charge of quality control, interpretation of the data and manuscript language editing. HZ was in charge of study coordination and quality control, and manuscript editing. WL contributed to fieldwork data collection and manuscript drafting.

## Conflict of Interest Statement

The authors declare that the research was conducted in the absence of any commercial or financial relationships that could be construed as a potential conflict of interest.

## Footnotes

1 ftp://ftp.fao.org/fi/stat/summary/a-6.pdf

2 http://sourceforge.net/projects/seqclean/

3 https://www.mothur.org

4 http://www.mothur.org/wiki/Align.seqs

5 http://www.mothur.org/wiki/Cluster

6 http://www.mothur.org/wiki/Classify.seqs

7 http://csbl.bmb.uga.edu/dbCAN/

8 http://www.uniprot.org/uniprot/

9 http://www.R-project.org

10 http://ieg2.ou.edu/MENA
