# Supplementary material for: Succession and Fermentation Products of Grass Carp (Ctenopharyngodon idellus) Hindgut Microbiota in Response to an Extreme Dietary Shift
Source: Front Microbiol. 2017 Aug 21;8:1585. doi: 10.3389/fmicb.2017.01585 (PMC5566599; doi:10.3389/fmicb.2017.01585)
Supplement: Supplementary file 1 [file Presentation_1.PDF]

Supplementary Information

Succession and fermentation products of grass carp (*Ctenopharyngodon idellus*) hindgut microbiota in response to an extreme dietary shift

Yaotong Hao[1,2], Shangong Wu[1,3*], Fan Xiong[1,3], Ngoc Tuan Tran[1], Ivan Jakovlić[4], Hong Zou[1], Wenxiang Li[1,3] and Guitang Wang[1,3*]

[1]*Key Laboratory of Aquaculture Disease Control, Ministry of Agriculture, and State Key Laboratory of Freshwater Ecology and Biotechnology, Institute of Hydrobiology, Chinese Academy of Sciences, Wuhan, 430072, P. R. China;*

[2]*Ocean College of Hebei Agricultural University, Qinhuangdao, 066003, P. R. China;*

[3]*University of Chinese Academy of Sciences, Beijing, 100039, P. R. China;*

[4]*Bio-transduction lab, Wuhan Institute of Biotechnology, Wuhan, Hubei Province, 430072, P. R. China.*

*Correspondence:

Shangong Wu, Fax: 86-027-68780123, E-mail: wusgz@ihb.ac.cn; and Guitang Wang, Fax: 86-027-68780123, E-mail: gtwang@ihb.ac.cn.

Summary: The supplementary materials, as shown below, include two supplementary texts, tree tables and ten figures.

Texts

Text S1. The detials of qPCR

Plasmid standards for qPCR assays were prepared from representative clones. Plasmids were purified and quantified by A260 measurement. To prepare the standard curve, the plasmids were gradient diluted with sterilized water. PCRs were performed in 96-well plates using a Bio-Rad CFX 96 Real-Time Detection System (Bio-Rad, Hercules, CA, USA). Each qPCR mixture contained 12.5 μL of 2×QuantiFast SYBR Green PCR Master Mix (QIAGEN, Hilden, Germany), 2 μL of DNA template and 2.5 μL of each primer (1 μM final concentration) and RNase-free water to a final volume of 25 μL. For every primer pair, triplicate qPCR reactions were assayed for each sample.

Text S2. *Bacteroides* spp. used in this study

The genomes of *Bacteroides dorei* HS1_L1_B_010, *B. dorei* HS1_L3_B_079, *B. fragilis* 638R, *B. fragilis* BOB25, *B. fragilis* NCTC 9343, *B. fragilis* YCH46, *B. helcogenes* P 36-108 ATCC 35417, *B. salanitronis* DSM 18170, *B. sp.* CF50, *B. thetaiotaomicron* VPI-5482, *B. vulgatus* ATCC 8482 and *B. xylanisolvens* XB1A were included to determine GH and PL families.

Tables

Table S1. Primers and amplification conditions for qPCR assays in this study

Target organism	Primers	Sequence (5’ to 3’)	References	Annealing temp (°C)
Prokaryote	PRK341F	CCTACGGGRBGCASCAG	Yu et al. (2005)	Initial denaturing at 95°C for 10 min; 40 cycles of denaturing at 95°C for 30 s, annealing at 60°C for 30 s, and extension at 72°C for 1 min.
	PRK806R	GGACTACYVGGGTATCTAAT		
Archaea	Arch787F	ATTAGATACCCSBGTAGTCC	Yu et al. (2005)	Initial denaturing at 95°C for 10 min; 40 cycles of denaturing at 95°C for 30 s, annealing at 60°C for 30 s, and extension at 72°C for 1 min.
	Arch1059R	GCCATGCACCWCCTCT		
Bacteria	BAC338F	ACTCCTACGGGAGGCAG	Stevenson & Weimer (2007)	Initial denaturing at 95°C for 10 min; 40 cycles of denaturing at 95°C for 30 s, annealing at 60°C for 30 s, and extension at 72°C for 1 min.
	BAC805R	GACTACCAGGGTATCTAATCC		
Bacteroidetes	Bact934F	GGARCATGTGGTTTAATTCGATGAT	Guo et al. (2008)	Initial denaturing at 95°C for 2 min; 40 cycles of denaturing at 95°C for 15 s, and annealing/extension at 60°C for 1 min.
	Bact1060R	AGCTGACGACAACCATGCAG		
Bacteroides	Bac386F	CTGAACCAGCCAAGTAGCG	Liu et al. (2003); Pang et al. (2005)	Initial denaturing at 95°C for 10 min; 40 cycles of denaturing at 95°C for 30 s, annealing at 60°C for 30 s, and extension at 72°C for 1 min.
	Bac593R	CCGCAAACTTTCACAACTGACTTA		
Firmicutes	Firm934F	GGAGYATGTGGTTTAATTCGAAGCA	Guo et al. (2008)	Initial denaturing at 95°C for 2 min; 40 cycles of denaturing at 95°C for 15 s, and annealing/extension at 60°C for 1 min.
	Firm1060R	AGCTGACGACAACCATGCAC		
Lachnospiraceae	Ccocc1F	CGGTACCTGACTAAGAAGC	Frank et al. (2007)	Initial denaturing at 95°C for 10 min; 45 cycles of denaturing at 95°C for 15 s, and annealing at 56°C for 15 s, and extension at 60°C for 30 s.
	Ccocc1R	AGTTTYATTCTTGCGAACG		

Table S2. Details of grass carp gut sample information

ID	Diet	Days of feeding	Sampling Time	Raw sequences *	Unique sequences *	OTU
1	Fish meal	0	16/09/2012	29282	17849	850
2	Fish meal	0	16/09/2012	26469	16713	735
3	Fish meal	0	16/09/2012	24389	14782	706
4	Sudan grass	1	17/09/2012	26458	21732	849
5	Sudan grass	1	17/09/2012	28597	22420	734
6	Sudan grass	1	17/09/2012	26318	21332	1021
7	Sudan grass	3	19/09/2012	26944	22017	1050
8	Sudan grass	3	19/09/2012	27760	22073	943
9	Sudan grass	3	19/09/2012	25708	17864	1111
10	Sudan grass	7	23/09/2012	25291	19625	1089
11	Sudan grass	7	23/09/2012	28229	20305	1085
12	Sudan grass	7	23/09/2012	23395	16753	1013
13	Sudan grass	11	27/09/2012	24206	19749	938
14	Sudan grass	11	27/09/2012	27263	19531	1146
15	Sudan grass	11	27/09/2012	20597	15197	910
16	Sudan grass	19	05/10/2012	24784	18587	983
17	Sudan grass	19	05/10/2012	21996	17178	859
18	Sudan grass	19	05/10/2012	22889	17103	959
19	Sudan grass	25	11/10/2012	21890	17883	963
20	Sudan grass	25	11/10/2012	25319	19795	934
21	Sudan grass	25	11/10/2012	23287	17074	1142
22	Sudan grass	33	19/10/2012	23548	17119	1011
23	Sudan grass	33	19/10/2012	24201	16714	1063
24	Sudan grass	33	19/10/2012	25751	17614	1104

* Raw sequences are the original data before filtering.
* Unique sequences are the remaining data after filtering.

Table S3. Peptidase families in *Cetobacterium somerae* ATCC BAA-474 based on UniProtKB

**Protein name**	**Enzyme Commission number**	**Family**	**Organism**
Lipoprotein signal peptidase	EC 3.4.23.36	Peptidase A8 family	*C. somerae* ATCC BAA-474
Pyrrolidone-carboxylate peptidase	EC 3.4.19.3	Peptidase C15 family	
LD-carboxypeptidase	-	-	
Peptidase M16 inactive domain protein	-	Peptidase M16 family	
Aminopeptidase	EC 3.4.11.-	Peptidase M18 family	
Leucyl aminopeptidase	EC 3.4.11.1	Peptidase M17 family	
Tripeptide aminopeptidase	EC 3.4.11.4	Peptidase M20B family	
Peptidase	-	Peptidase M23 family	
Methionine aminopeptidase	EC 3.4.11.18	Peptidase M24A family, Methionine aminopeptidase type 1 subfamily	
Uncharacterized protein	-	Peptidase M24B family	
Carboxypeptidase Taq metallopeptidase	-	Peptidase M32 family	
ATP-dependent zinc metalloprotease FtsH	EC 3.4.24.-	Peptidase M41 family	
Protease HtpX homolog	EC 3.4.24.-	Peptidase M48B family	
RIP metalloprotease RseP	-	Peptidase M50 family	
Serine-type D-Ala-D-Ala carboxypeptidase	-	Peptidase S11 family	
ATP-dependent Clp protease proteolytic subunit (Endopeptidase Clp)	EC 3.4.21.92	Peptidase S14 family	
Xaa-Pro dipeptidyl-peptidase	EC 3.4.14.11	peptidase S15 family	
Lon protease	EC 3.4.21.53	Peptidase S16 family	
Signal peptidase I	EC 3.4.21.89	Peptidase S26 family	

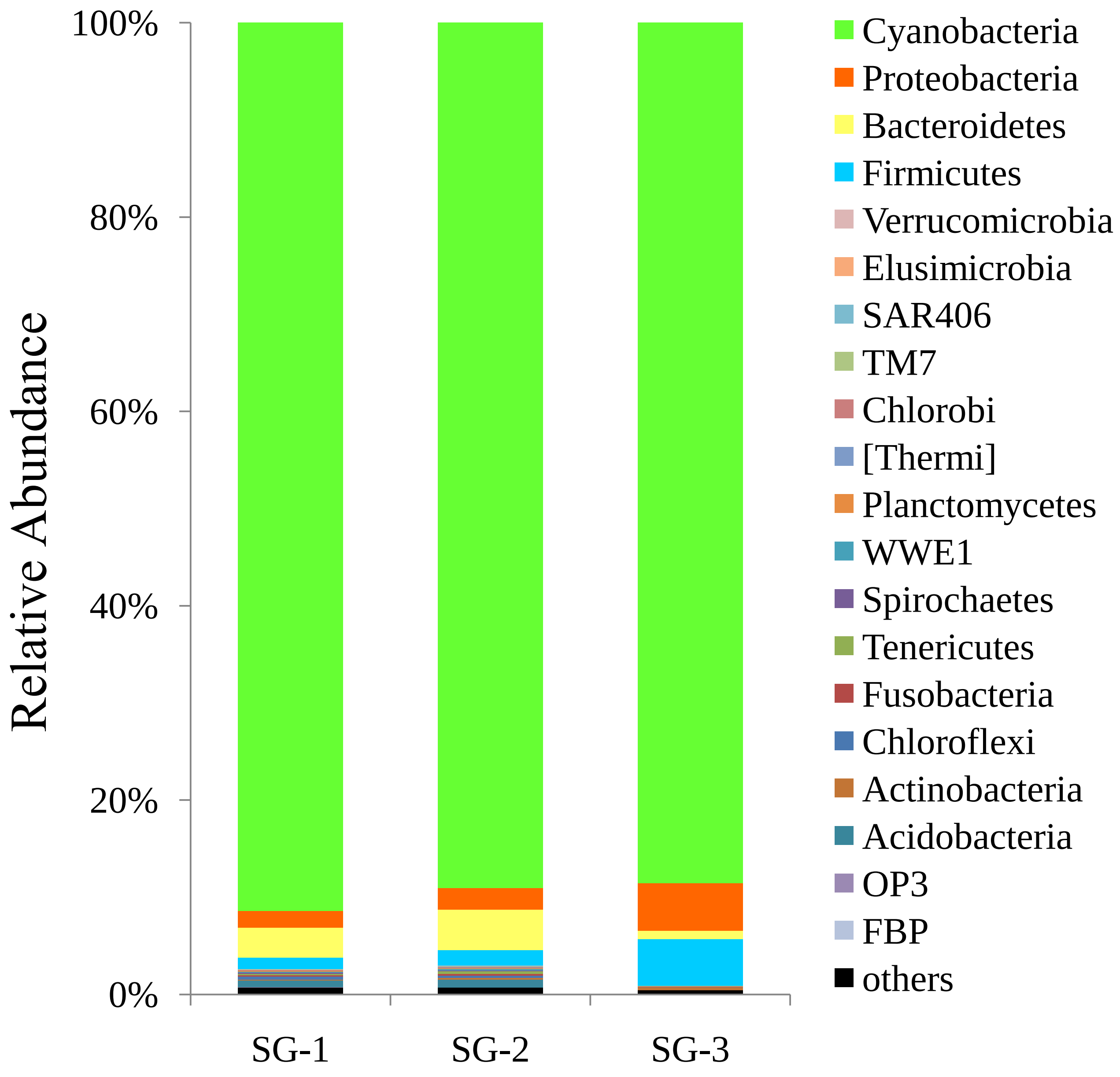

Figure S1: Relative abundance of different phyla in Sudan grass. The top 20 phyla of bacteria are shown.

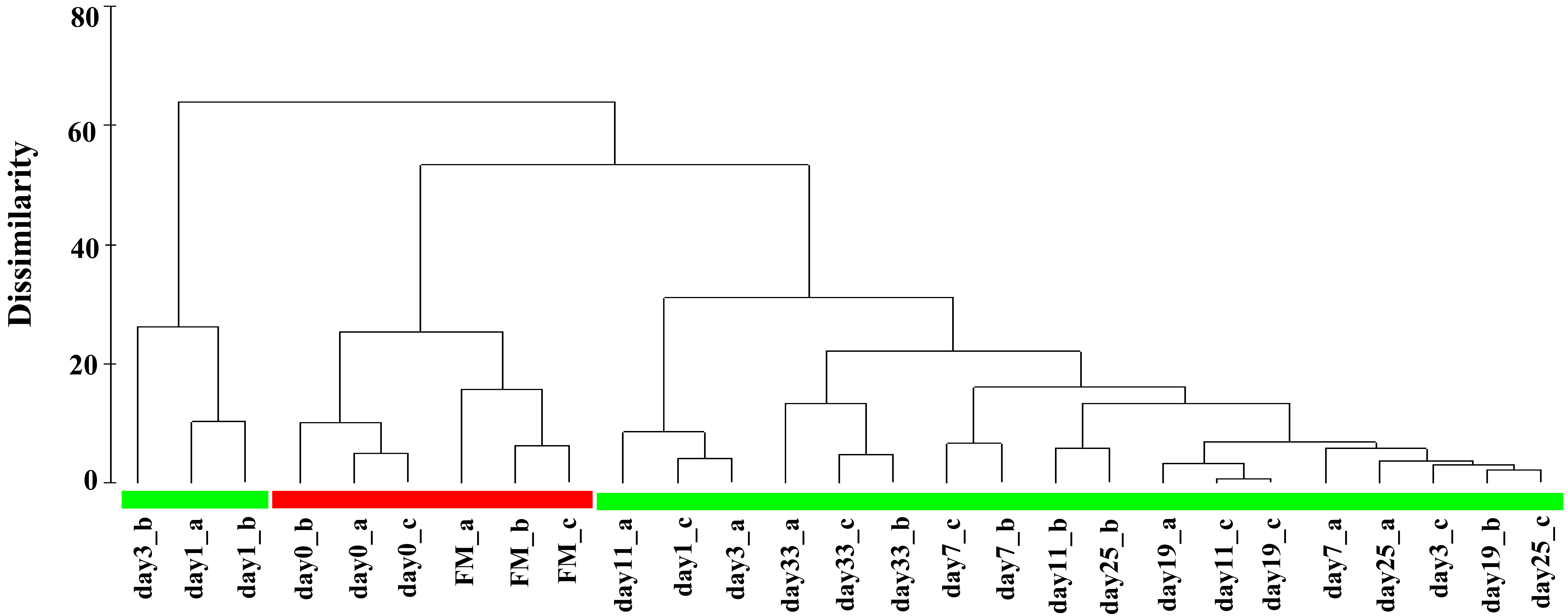

Figure S2: Cluster analysis of all samples based on Bray-Curtis distance. Samples of FM_a, FM_b and FM_c are from Wu et al. (2015).

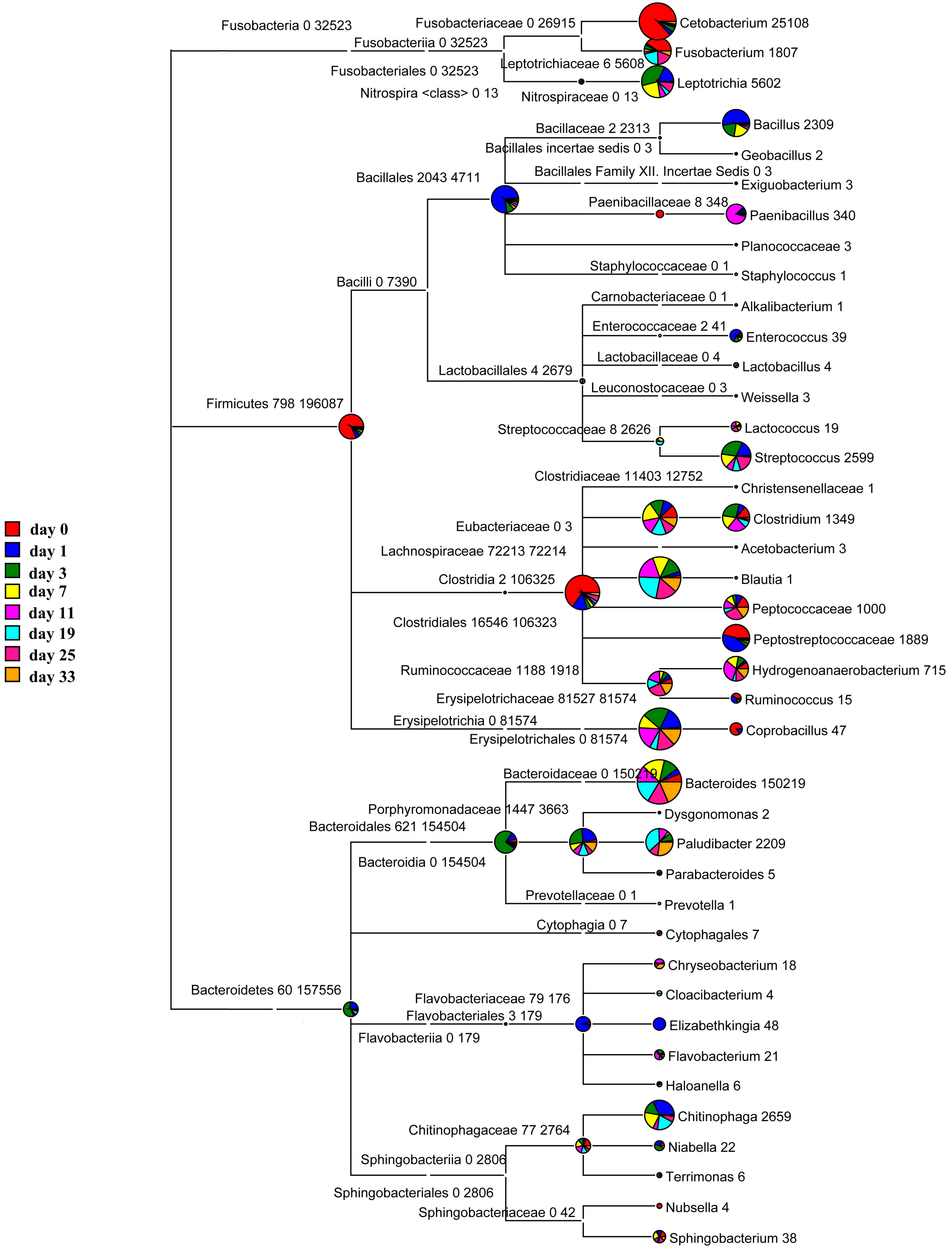

Figure S3: Taxonomic composition of different samples showed with the phylogenetic tree of Fusobacteria, Firmicutes, and Bacteriodetes. Taxon name except the lowest one is followed by two numbers. The first number represents the number of sequences belonging to this taxon but not belonging to its lower taxa; the second number represents all the number of sequences belonging to this taxon and all its lower taxa. The area of the pie chart represents the relative abundance of each sample.

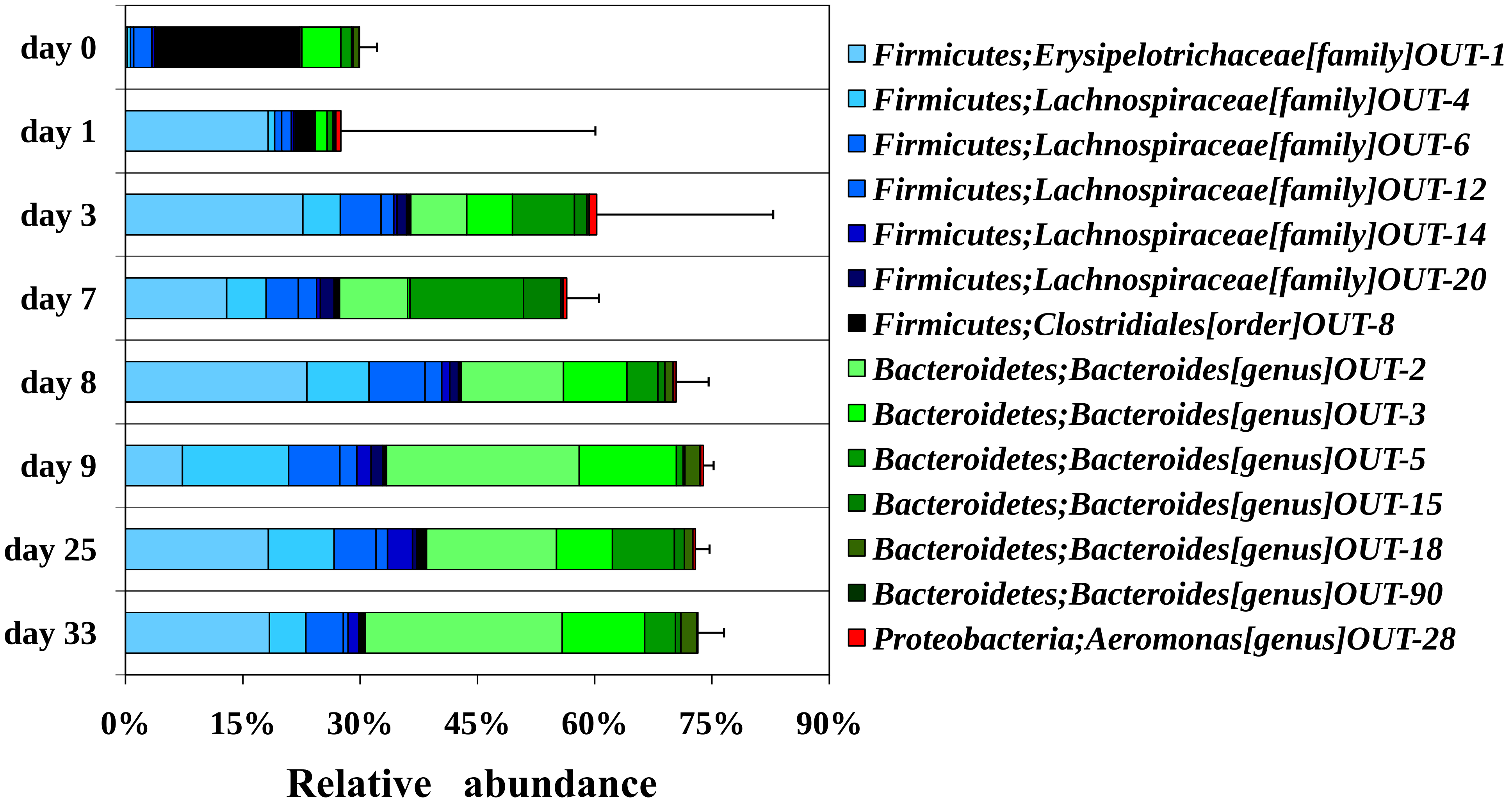

Figure S4: Relative abundance of 14 OTUs shared by all grass carp hindgut samples.

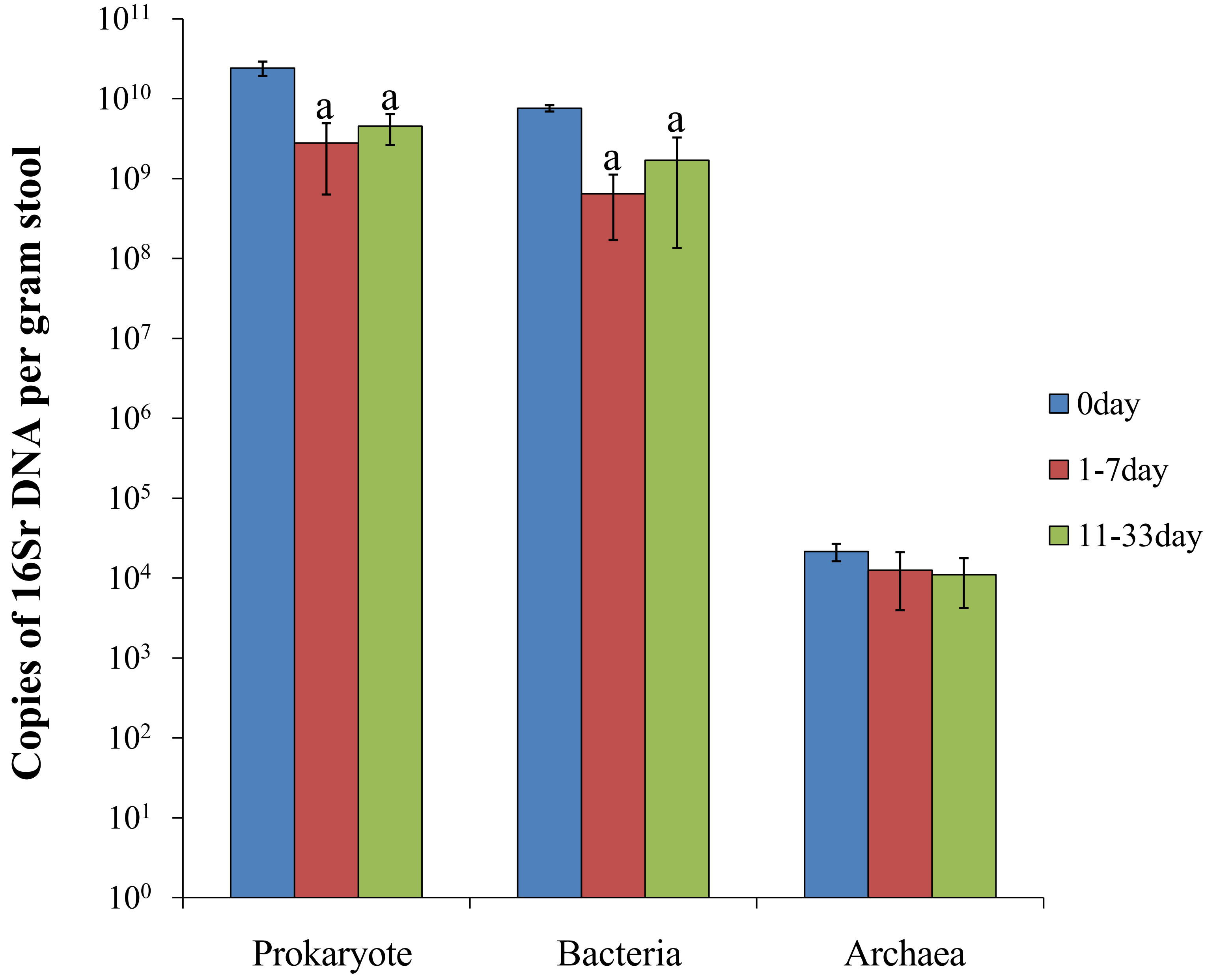

Figure S5: The 16S rDNA copies of prokaryote, bacteria and archaea quantified by qPCR. Error bars represent the standard error of the medians. Student T-test was used to analyze the differences between different stages of dietary shift. “a” means a significant difference between day 0 and plant-based samples, $P < 0.05$.

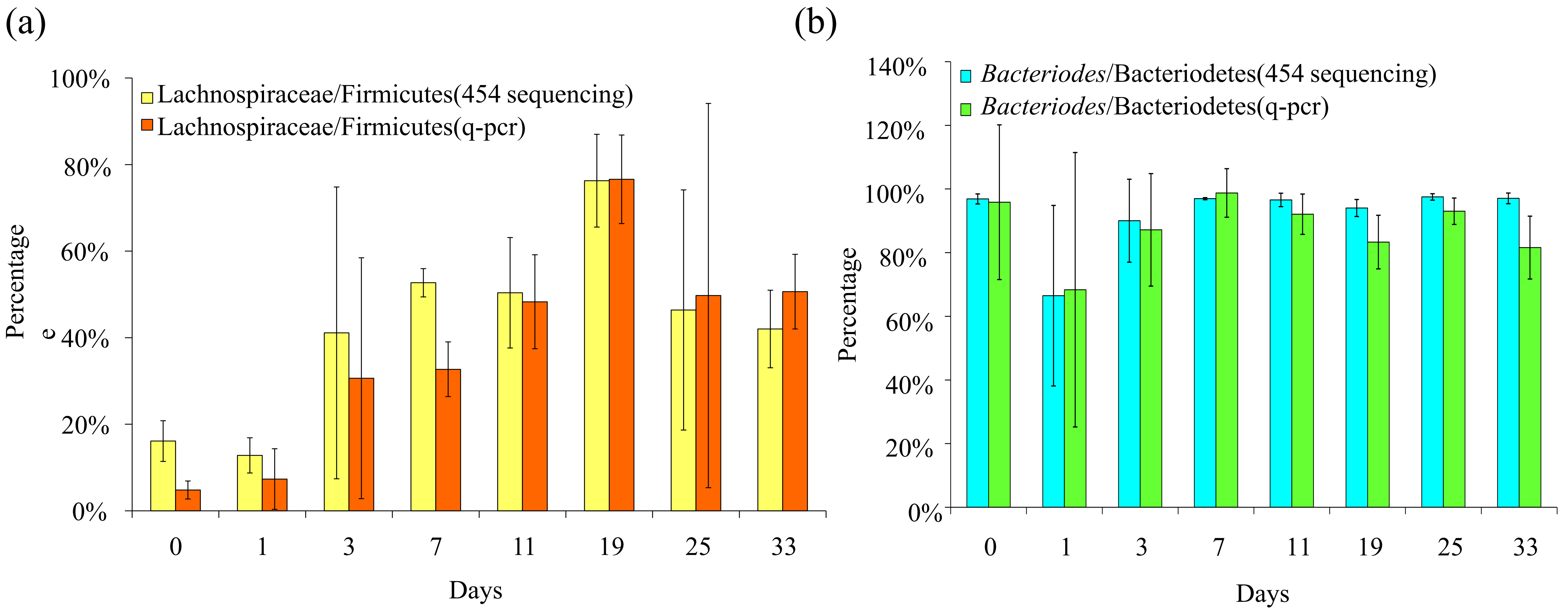

Figure S6: The ratios of Lachnospiraceae/Firmicutes (a) and Bacteroides/Bacteroidetes (b) determined by 454 sequencing and qPCR.

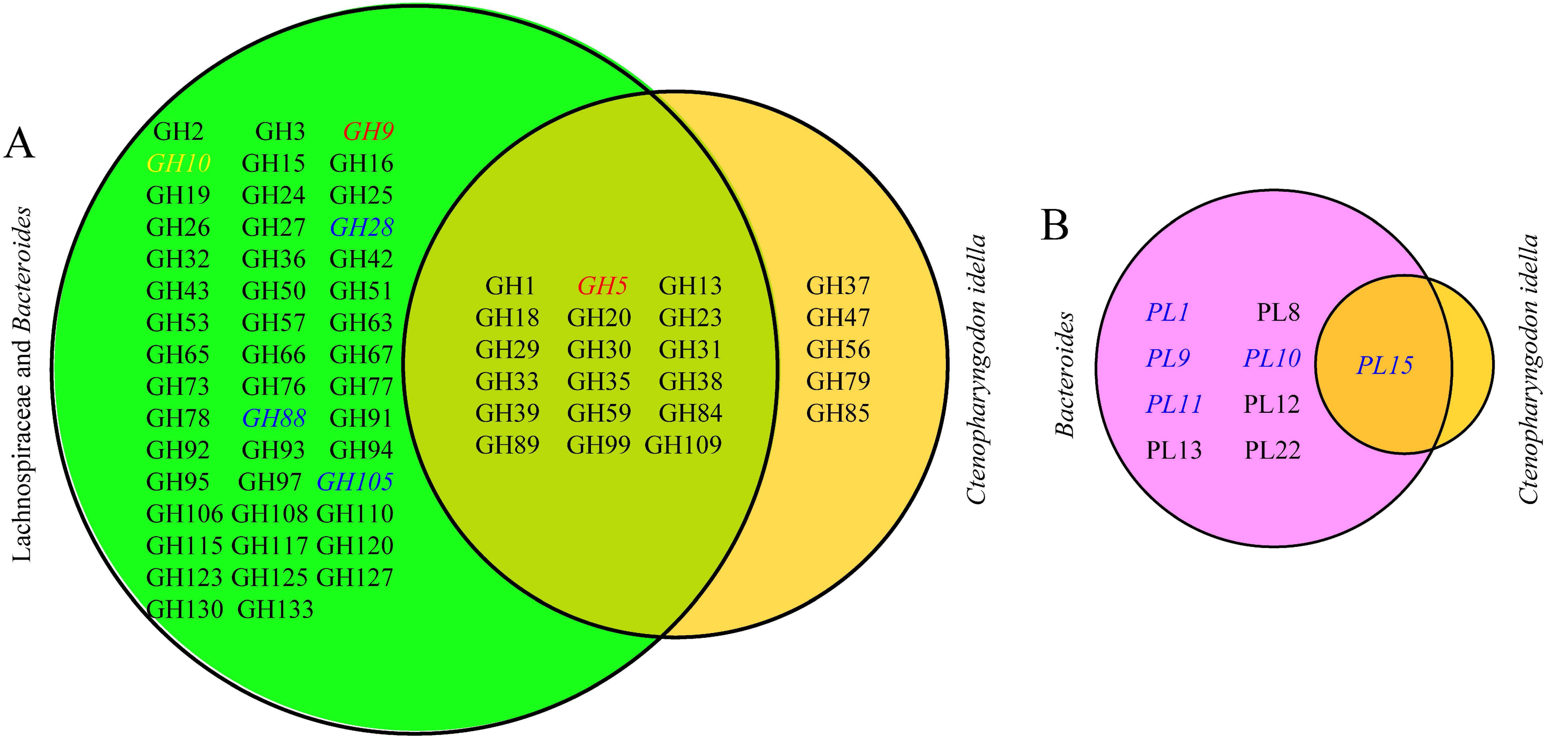

Figure S7: Venn diagrams showing the Carbohydrate-Active enZymes (CAZy) families in grass carp, Bacteroides, and Lachnospiraceae. (A) The comparison of GH families between grass carp and bacterial groups of Bacteroides and Lachnospiraceae. (B) The comparison of PL families between grass carp and Bacteroides. The CAZy families highlighted in red are associated with cellulase-encoding genes, in yellow are associated with xylanase-encoding genes and in blue are associated with pectinase encoding genes. GH5 in bacteria is cellulase, but it is β-glucuronidase in grass carp; PL15 in bacteria is pectinase, but it is dermatan-sulfate epimerase in grass carp.

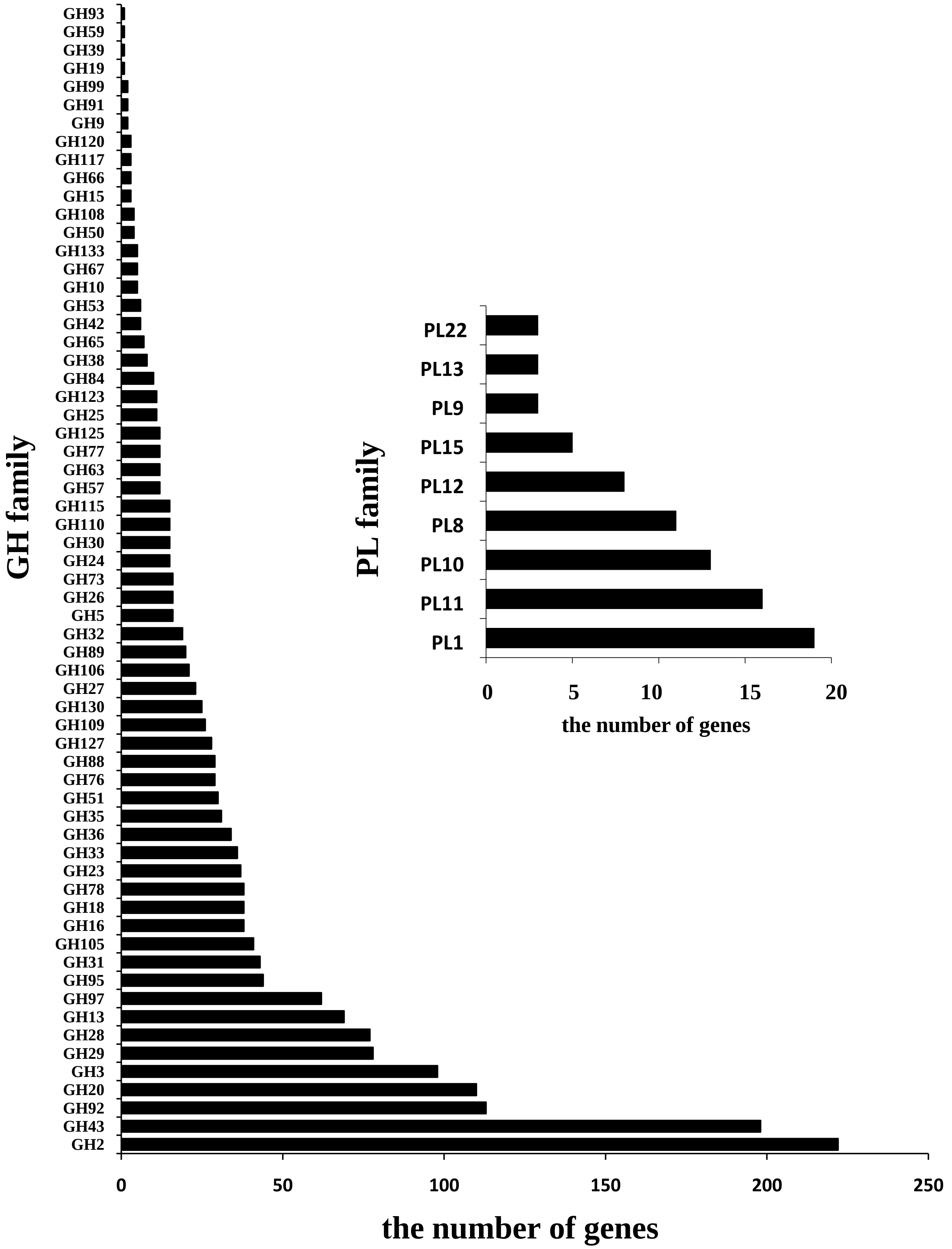

Figure S8: The numbers of GH and PL encoding genes in Bacteroides.

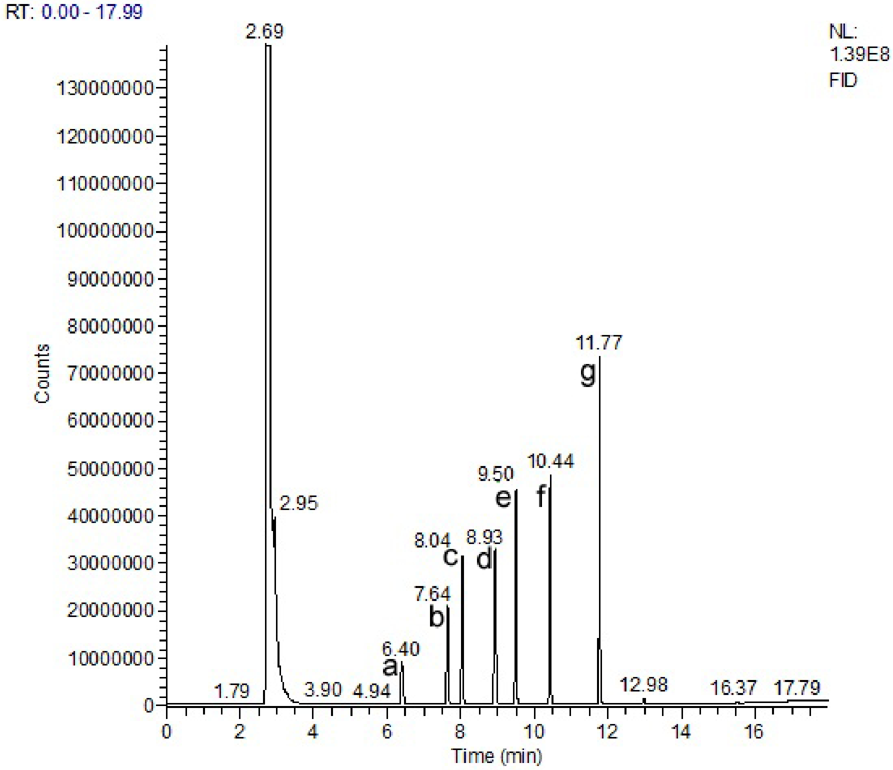

Figure S9: Gas chromatograph analysis of standards. a: acetic acid, b: propionic acid, c: n- butyric acid, d: i-butyric acid, e: n-valeric acid, f: i-valeric acid, g: n-caproic acid.

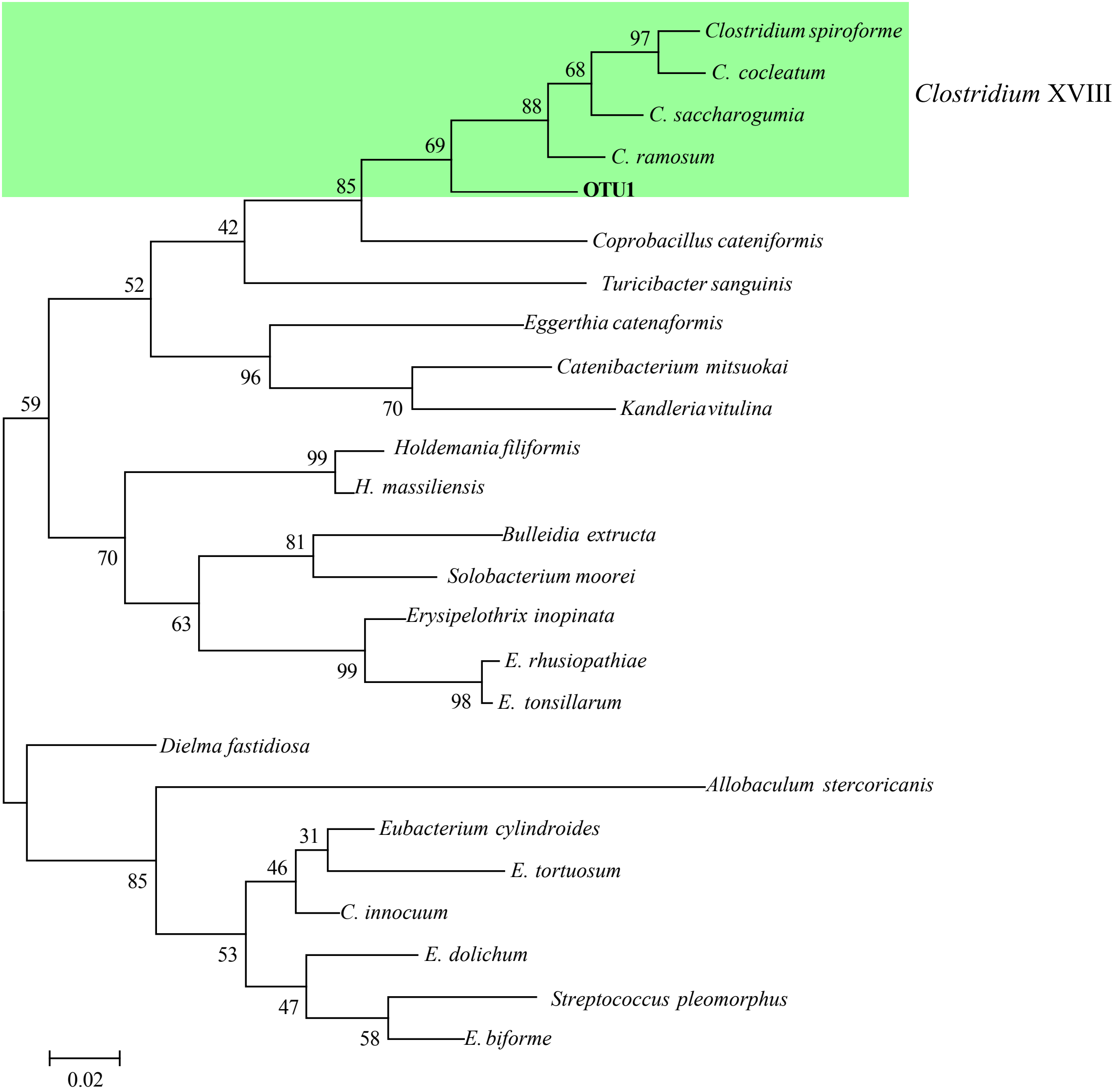

Figure S10: Phylogenetic tree of Erysipelotrichaceae. In addition to the representative sequence of OTU1, the other 16S rDNA sequences in the family of Erysipelotrichaceae are downloaded from GenBank database. All these 16S rDNA sequences are aligned together and the phylogenetic tree is constructed by the maximum likelihood method.

References of supplementary materials

Frank DN, Amand ALS, Feldman RA, Boedeker EC, Harpaz N, Pace NR. (2007) Molecular-phylogenetic characterization of microbial community imbalances in human inflammatory bowel diseases. Proc Natl Acad Sci USA **104:** 13780-13785.

Guo X, Xia X, Tang R, Zhou J, Zhao H, Wang K. (2008) Development of a real-time PCR method for Firmicutes and Bacteroidetes in faeces and its application to quantify intestinal population of obese and lean pigs. Lett Appl Microbiol **47:**367-373.

Liu C, Song Y, McTeague M, Vu AW, Wexler H, Finegold SM. (2003) Rapid identification of the species of the *Bacteroides fragilis* group by multiplex PCR assays using group-and species-specific primers. FEMS Microbiol Lett **222:** 9-16.

Pang X, Ding D, Wei G, Zhang M, Wang L, Zhao L. (2005) Molecular profiling of *Bacteroides* spp. in human feces by PCR-temperature gradient gel electrophoresis. J Microbiol Meth **61:** 413-417.

Stevenson DM, Weimer PJ. (2007) Dominance of *Prevotella* and low abundance of classical ruminal bacterial species in the bovine rumen revealed by relative quantification real-time PCR. Appl Microbiol Biotechnol **75:**165-174.

Yu Y, Lee C, Kim J, Hwang S. (2005) Group-specific primer and probe sets to detect methanogenic communities using quantitative real-time polymerase chain reaction. Biotechnol Bioeng **89:** 670-679.

Wu SG, Ren Y, Peng C, Hao YT, Xiong F, Wang GT, Li WX, Zou H, Angert ER. (2015) Metatranscriptomic discovery of plant biomass-degrading capacity from grass carp intestinal microbiomes. FEMS Microbiol. Ecol. **91:** fiv107.
